# A Novel Method Based on Headspace-Ion Mobility Spectrometry for the Detection and Discrimination of Different Petroleum Derived Products in Seawater

**DOI:** 10.3390/s21062151

**Published:** 2021-03-19

**Authors:** Lucas Jaén-González, Ma José Aliaño-González, Marta Ferreiro-González, Gerardo F. Barbero, Miguel Palma

**Affiliations:** Department of Analytical Chemistry, Faculty of Sciences, IVAGRO, University of Cadiz, 11510 Cadiz, Spain; lucas.jaengonzalez@alum.uca.es (L.J.-G.); marta.ferreiro@uca.es (M.F.-G.); gerardo.fernandez@uca.es (G.F.B.); miguel.palma@uca.es (M.P.)

**Keywords:** petroleum-derived products, seawater, spills, ion mobility spectrometry, IMS sum spectrum, chemometric, sensors, detection, discrimination

## Abstract

The objective of the present study is to develop an optimized method where headspace-ion mobility spectrometry is applied for the detection and discrimination between four petroleum-derived products (PDPs) in water. A Box–Behnken design with a response surface methodology was used, and five variables (incubation temperature, incubation time, agitation, sample volume, and injection volume) with influences on the ion mobility spectrometry (IMS) response were optimized. An IMS detector was used as a multiple sensor device, in which, each drift time acts as a specific sensor. In this way, the total intensity at each drift time is equivalent to multiple sensor signals. According to our results, 2.5 mL of sample incubated for 5 min at 31 °C, agitated at 750 rpm, and with an injection volume of 0.91 mL were the optimal conditions for successful detection and discrimination of the PDPs. The developed method has exhibited good intermediate precision and repeatability with a coefficient of variation lower than 5%, (RSD (Relative Standard Deviation): 2.35% and 3.09%, respectively). Subsequently, the method was applied in the context of the detection and discrimination of petroleum-derived products added to water samples at low concentration levels (2 µL·L^−1^). Finally, the new method was applied to determine the presence of petroleum-derived products in seawater samples.

## 1. Introduction

Different technological and industrial developments over the past decades have brought about an increment in petroleum exploitation as well as the materialization of new petroleum-derived products (PDPs) [1]. The number of extraction, transport, or treatment processes where petroleum is involved has, therefore, increased noticeably, and as a consequence, oil and fuel spills have become more frequent [2,3,4]. Spills may be a consequence of the accidental collision of crude oil tankers or undesired leaks from underwater pipelines, among other reasons. However, they may also be a consequence of intentional inappropriate waste management by refineries or other industries, or even the result of negligent practices, such as inadequate tank cleaning procedures in an attempt to avoid cleaning taxes [5,6,7].

PDPs contain thousands of hazardous chemicals that pose a potential threat to flora, fauna, and human health [8,9]. Additionally, the natural processes that degrade them require very long times to reach completion because of PDP’s complex chemical composition and physical structure. This means that they remain in the environment for lengthy periods of time. Chemical toxicity is not the only serious consequence of spills, most PDPs exhibit dense and opaque physical characteristics so that, when on the sea surface, they prevent sunlight from reaching the biological marine environment, which represents a clear threat to sea-living creatures [10]. Furthermore, part of the water used in agriculture is recycled water from PDP polluted sources, which means that a proportion of these polluting substances could be absorbed by agricultural produce, and later on, be ingested by animals or people with harmful consequences [11,12].

The negative consequences of spills have made of them a growing concern for society and, therefore, different control and removal methods have been developed to minimize their impact on the environment [13,14]. The first stage of spill control consists of containment and recovery, followed by strategies that focus on shoreline cleaning and elimination of any remaining oil/fuel [15]. For these latter purposes, chemical dispersants or physical methods such as compression or centrifugation are employed [16,17]. However, the correct identification of the PDPs involved in each particular spill is a key factor in ensuring the correct management. The fast and accurate identification of the specific PDPs involved brings about two important advantages. Firstly, this allows for the correct selection of the removal method in order to minimize toxicity, exposure and the remaining amount. Secondly, it allows for the tracing of the spill to a particular source, which contributes to avoiding future spills, as well as to the implementation of the necessary legal measures, when applicable [18,19].

PDPs are a mixture of primarily aliphatic, aromatic hydrocarbons and heterocycles that come from crude oil [20,21,22]. Although they all come from a common source, the different refining processes that they undergo (cracking, alkylation, catalytic reforming, or distillation) may drastically change their composition. Additionally, a number of additives are added at different stages in order to modify their chemical and/or physical properties and, finally, some of their fractions may be blended to elaborate specific commercial products of interest. All of these factors make PDP difficult compounds to successfully identify by means of ordinary forensic methods.

Most of the methods developed for the identification and discrimination of PDPs in water are based on two main analytical techniques: (i) gas chromatography (GC) [23,24,25,26], which provides information after the separation of the individual chemical compounds, and (ii) infrared spectroscopy (IR) [27,28], which permits identification based on the whole spectra of the PDP samples. Both techniques are based on the identification of individual compounds and they been proven to be effective for this purpose. Nowadays, the use of fingerprints represents an important improvement in environmental research studies [29,30,31,32,33], since the methodologies that have been developed are rapid, reliable, and easy-to-use, which are essential characteristics to confront highly toxic events such as PDP spills. In fact, headspace-mass spectrometry (HS-MS), has been successfully used for the detection and discrimination of PDPs that have suffered a weathering process [34,35] or PDPs in water samples [29].

On the other hand, ion mobility spectrometry (IMS) has been successfully applied for a large variety of purposes, such as food fraud detection [36,37,38,39], fire debris analysis [40,41], and also to uncover drugs and explosives [42,43,44]. As regards environmental issues, IMS has mainly been used to detect bacterial contamination [45,46,47], to characterize biodegraded PDPs [48] and, in combination with extraction techniques, to identify polycyclic aromatic hydrocarbons in water [49,50]. IMS exhibits a very low limit of detection (within the µL·L^−1^ range), and it does not require complex sample preparation methods [51]. It is usually coupled to headspace (HS) techniques and the methods based on this analytical technique do not usually require solvents, thus, their residues are minimal and could be considered as environmentally friendly [52]. Lastly, IMS operates at atmospheric pressure, which means that IMS can be used for the real-time monitoring of the analysis procedures and, therefore, could be applied for the detection of PDPs spills in water. On the other hand, the IMS technique can be used as a multiple sensor device, in which each drift time in the detector acts as a specific “sensor” and the total volatile compounds intensity collected at each drift time is equivalent to multiple sensor signals [36,40,48,53,54,55].

The aim of this research is to develop a method based on HS-IMS for the detection and discrimination of PDPs in water. For this purpose, a Box–Behnken design (BBD) with response surface methodology (RSM) was applied to the five variables that were previously selected. IMS was used as a multiple sensor device with all the advantages that this entails. Ion mobility sum spectra (IMSS) has been proposed as a novel approach to determine the optimum conditions that are required for the best discrimination between different PDPs in water. The second goal of this investigation consists of applying the developed method, i.e., IMSS combined with certain chemometric tools to PDP-added water samples in order to determine how suitable it is for detecting and discriminating PDP spills in seawater samples in a reliable, rapid, and easy-to-use manner.

## 2. Materials and Methods

### 2.1. Samples

#### 2.1.1. Water Samples

Water samples of different origin and type (seawater and freshwater) were analyzed by HS-GC-IMS and their blank spectra were obtained. For this reason, no differences between fresh water and seawater were considered. The seawater both for the pure water samples, as well as for the PDP-added samples used to optimize the method, were collected from Cadiz Bay (Valdelagrana Beach). Two 500 mL replicas of pure seawater were collected in opaque jars and kept at 4 °C until analysis. Finally, the optimized method was tested on unaltered seawater samples collected from different points at the beach shores of the Cadiz Bay. The locations of seawater collection spots are included in Table 1. The preservation conditions of the unaltered seawater samples were the same as for the initial pure water used for the optimization of the process.

#### 2.1.2. PDP Samples

The main PDPs that are most frequently found in industrial and seaport spills were added to the water samples in order to optimize the detection and discrimination of the method. A total of 16 PDPs that could classified into 4 different groups were selected: gasoline (*n* = 4), diesel (*n* = 4), lubricant (*n* = 4), and kerosene (*n* = 4). A description of the sample sources can be seen in Table 2. It can be observed that the samples were collected on different dates and from different places in order to ensure a wide variety of samples. Their physical and chemical properties were previously determined by our research team in order to correctly classify them into the appropriate group [56].

#### 2.1.3. Petroleum Products in Water Samples

For the optimization of a method to discriminate between different PDPs in water, four 50 µL·L^−1^ solutions using Gas_95_1, Dies_1, LUB_1 and Ker_1 to ensure the discrimination between the four groups were prepared. According to Spanish law (Royal Decree 60/2011 by the *Spanish Ministry of the Environment Rural and Marine Affairs*), the maximum level of PDP content in water should not exceed 8 µL·L^−1^ to guarantee a safe environment [57]. For this reason, and in order to ensure successful discrimination, much higher concentrations (50 µL·L^−1^) were used for the optimization process. Later on, in order to test the efficacy of the developed method, a number of PDP-added samples were elaborated by adding 16 different PDPs to pure water samples at 8, 4, 2, 0.8, and 0.4 µL·L^−1^ concentration levels.

### 2.2. HS-GC-IMS Acquisition

The pure water and the PDP-added water samples were analyzed by headspace-gas chromatography-ion mobility spectrometry (HS-GC-IMS) Flavour Spec (G.A.S., Dortmund, Germany). No pretreatment was applied to vials, instead, they were immediately placed into the autosampler oven. ^3^H Tritium beta radiation was the ionization methodology used and a nitrogen generator (G.A.S., Dortmund, Germany) was selected to provide the 99.999% pure nitrogen employed as the drift and carrier gas. The GC column was a 20 cm multicapillary MCC OV-5 (G.A.S., Dortmund, Germany).

A Box–Behnken design was applied to optimize the conditions related to HS. Refer to Section 2.2.1. for selection and description.

The GC-IMS conditions were as follows: EPC1 (drift gas) was fixed at top flow (250 mL/min) to avoid the noise from no-ionized compounds in the analysis. EPC2 (carried gas) was set according to the following ramp: 5 mL/min (t = 0 min), 10 mL/min (t = 5 min), and 25 mL/min (t = 10 min). Total analysis time: 15 min. The system temperature was set as follows: T_1_: 45 °C; T_2_: +5 °C over the HS temperature; T_3_: 80 °C and T_4_: 80 °C.

#### 2.2.1. Optimization of the Conditions

The different variables that affect the IMS spectra signal are mostly related to the particular conditions employed to generate the HS of the volatile compounds. According to the literature, the most influential variables are incubation temperature, incubation time, agitation, volume of injection, and volume of the sample [58]. Therefore, these were the variables that we optimized by means of a BBD with RSM. Each variable was analyzed at three different levels, as shown in Table 3, where (−1) represents low level, (0) middle level, and (1) high level.

The studied ranges were chosen according to the aim of this study: the development of a rapid analytical method to detect and discriminate PDPs in water samples. Thus, the incubation time was set between 5 and 25 min. The incubation temperature, agitation, and volume of injection were limited by the conditions allowed by the GC-IMS, and the sample volume was limited to 2.5 mL in order to achieve a balance between the reliable headspace and a short analysis time.

BBD, in conjunction with RSM, was applied to optimize the conditions in order to discriminate the PDPs in the water samples. The BBD design consisted of 46 experiments with 6 repetitions in the central point (Table A1). All the trials were performed in a random order.

### 2.3. Data Treatment

#### 2.3.1. IMS Sum Spectrum

A two-dimensional data matrix is usually obtained from a HS-GC-IMS analysis, where the GC information is represented on the Y-axis as the retention time, and the IMS information is shown on the X-axis as the drift time. This matrix provides fundamental information for the identification of individual compounds, but the data treatment is time-consuming and requires specific analytical skills. They are, therefore, difficult to use in routine control analyses, such as those typically performed in environmental forensic investigations.

**Figure 1:**
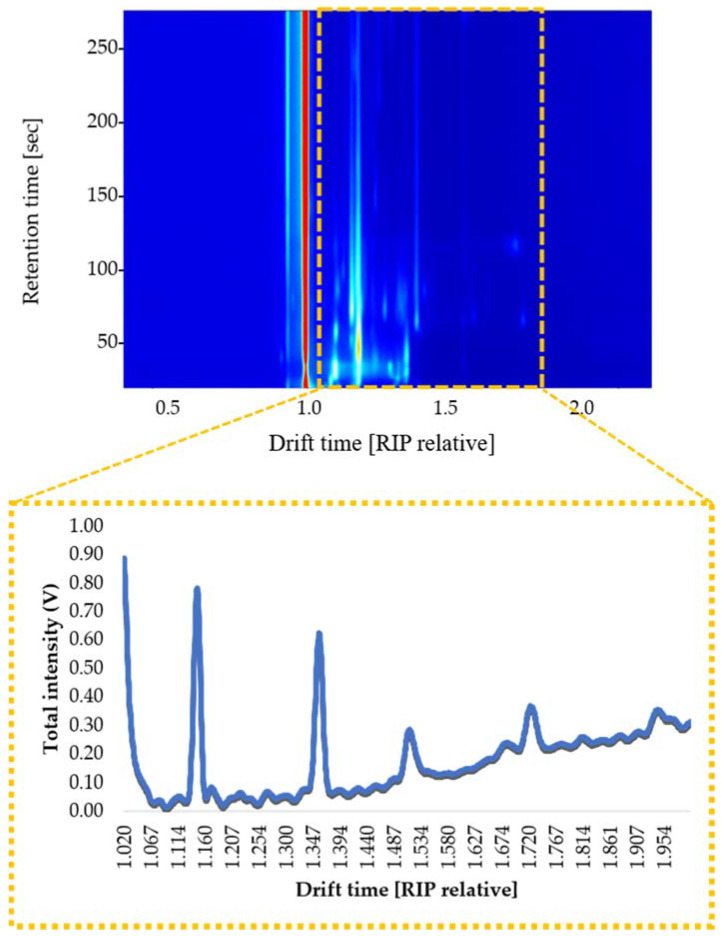
Topographic map of a gasoline-added water sample and ion mobility sum spectra (IMSS) obtained from the selected zone (yellow square).

For this reason, IMS sum spectrum (IMSS) has been proposed as an alternative. Initially, each compound’s drift time was normalized by means of the software application to the signal of the reaction ion peak (RIP). RIP is the signal produced by the water in the air that has been ionized by tritium beta radiation. It represents the total number of ions available for ionization, and therefore, it is used as the reference signal. Then, the total intensities at each drift time were equaled, assuming no chromatographic information was used, and the IMS detector acted as the sensor. The resulting IMSS includes the intensity levels corresponding to 4500 drift times, from 0.000 to 4.500 (RIP relative). The spectra have been reduced to the zone that includes the compounds of interest. This is why the IMSS were reduced from 1.020 to 2.000, with a total of 980 drift times (Figure 1). In all cases, the IMSSs were normalized by assigning one unit to the maximum intensity. The IMSS of all the samples was obtained by means of LAV HS-GC-IMS software (G.A.S., Dortmund, Germany).

#### 2.3.2. Data Analysis

Statgraphic Centurion XVI. I (Statgraphics Technologies, Inc., The Plains, VA, USA) was used for the development of the BBD-RSM, based on a total of 46 experiments, for the determination of the optimum conditions.

Once the optimum conditions were determined, a number of PDP-added samples, as well as pure water samples, were analyzed under such conditions. The IMSSs obtained from each one of the analyses, as well as the specific chemometric tools, were used to determine the useful information to detect and discriminate each PDP added to the samples. Thus, hierarchical cluster analysis (HCA) as a non-supervised methodology, and linear discriminant analysis (LDA) as a supervised tool, were carried out by means of the statistical computer package IBM SPSS Statistics 22 (Armonk, NY, USA).

## 3. Results and Discussion

### 3.1. Optimization of the Method

The first aim of this research was the optimization of a method based on IMSS to discriminate between different PDPs in water samples. Based on their impact on the HS-IMS responses, five variables were selected: incubation time, incubation temperature, agitation, injection volume, and sample volume. A BBD-RSM was applied to evaluate the effect from each variable on the response and to determine the optimum conditions for identifying the different PDPs in the water samples. Each sample’s IMSS characteristics were used for the optimization of the method to ensure the discrimination of the different PDPs added to the water samples, as already explained in Section 2.2.1.

It was observed that water samples do not produce any kind of signal, while the mixed PDP-added samples at 50 µL·L^−1^ produced specific signals in the detector. For this reason, it was decided that the purpose of the optimization was to maximize the differences between the responses from the four different types of PDP-added samples. Therefore, only the 46 experiments that were performed on these four sample types were applied the BBD.

For this purpose, gasoline, diesel, lubricant and kerosene solutions at 50 µL·L^−1^ in water were analyzed under the optimal conditions that had been previously determined for each one of the 46 experiments. An IMSS was obtained for each one of the 184 analyses (46 experiments × four solutions). These were normalized and reduced to the drift range where the compounds could be detected (from 1.020 to 2.000 (RIP relative)). Then, the differences between the IMSS response intensities at each one of the drift times were calculated. The sum of the drift time differences between Gas-Dies, Gas-Lub, Gas-Ker, Dies-Lub, Dies-Ker and Lub-Ker IMSS responses was used as the signal to be optimized.

An analysis of variance (ANOVA) was used to evaluate the influence from each factor and from the possible interactions between them on the successful discrimination of each one of the four PDPs in the water samples. The correlation between the actual differences in IMSS response intensities and the predicted values was evaluated by means of Equation (1).

Y = *β*_0_ + *β*_1_X_1_ + *β*_2_X_2_ + *β*_3_X_3_ + *β*_4_X_4_ + *β*_5_X_5_ + *β*_12_X_1_X_2_ + *β*_13_X_1_X_3_ + *β*_14_X_1_X_4_ + *β*_15_X_1_X_5_ + *β*_23_X_2_X_3_ + *β*_24_X_2_X_4_ + *β*_25_X_2_X_5_ + *β*_34_X_3_X_4_ + *β*_35_X_3_X_5_ + *β*_45_X_4_X_5_ + *β*_11_X_1_^2^ + *β*_22_X_2_^2^ + *β*_33_X_3_^2^ + *β*_44_X_4_^2^ + *β*_55_X_5_^2^ (1)

In this equation, Y is the predicted sum of the differences between the samples, which depends on a number of independent variables and quadratic coefficients. The variables are as follows: *β*_0_ is the model constant; X_1_ is the incubation time; X_2_ is the incubation temperature; X_3_ is the agitation; X_4_ is the injection volume; X_5_ is the sample volume. On the other hand, the coefficients are the following: *β*i is the linear coefficient and *β*ij is the cross-product coefficient. The coefficients of the different parameters in the quadratic polynomial equation, as well as their significance (*p*-values), are shown in Table 4.

The factors that exhibited a *p*-value lower than 0.05 were considered to be significant factors, since they affected the response at the selected level of significance (95%). In this case, the incubation temperature, sample volume, and the quadratic term of the sample volume were determined as the influential variables.

**Figure 2:**
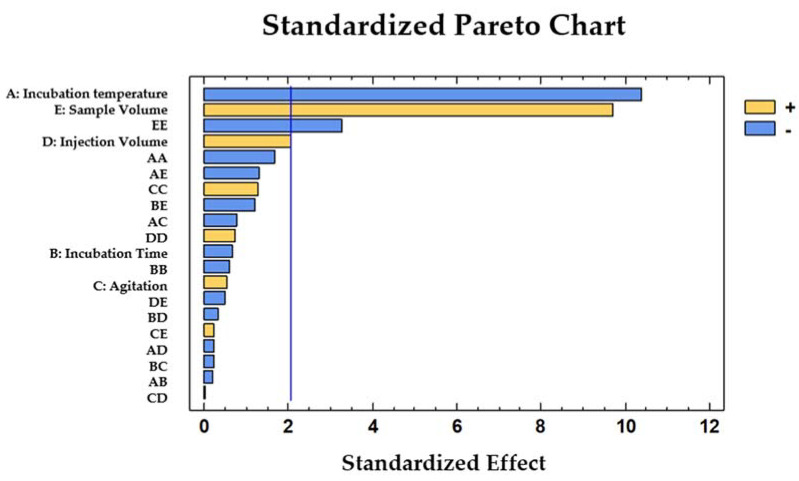
Standardized Pareto chart representing the five variables optimized for the discrimination between gasoline, diesel, lubricant and kerosene in water.

The incubation temperature presented a negative coefficient (b_1_ = −1294.850), which implies that the four groups were more efficiently discriminated when the incubation temperature was at the low limit of the studied range. On the other hand, the sample volume exhibited a positive coefficient (b_5_ = 1212.570), which means that the discrimination between the four groups was more successful when the sample volume was at the high limit of the studied range. The effect of the variables and their interaction on the response variable were visually represented in a standardized Pareto chart (Figure 2).

In order to evaluate the statistically significant agreement between the measured and the estimated response, the squared correlation coefficient (R^2^) was used and, in this case, a value of R^2^ = 0.903 was obtained. Furthermore, a lack-of-fit analysis of variance was conducted to determine the linearity of the model and the result was 0.91 (*F* = 0.95), which means that the developed model was linear and that the differences between the predicted and the actual values were not significant.

**Figure 3:**
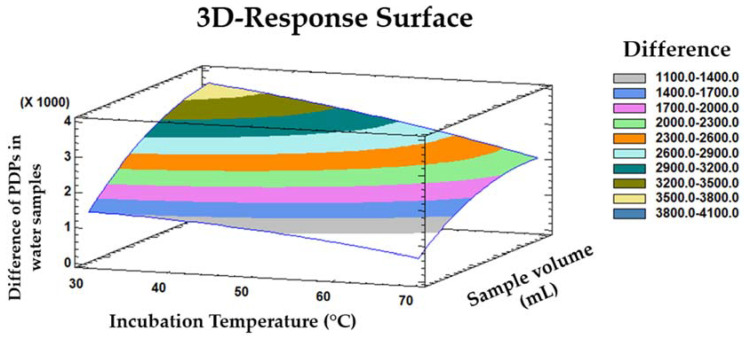
3D-surface plot representing the influence of incubation temperature and sample volume on the differences among the ion mobility spectrometry (IMS) intensities between PDPs in water.

Finally, a three-dimensional surface plot of the quadratic response (Figure 3) was produced by applying the polynomial equation (Equation (1)). The combined effect from two of the influential variables (namely, the incubation temperature and sample volume) on the differences between the quadratic response from the four types of PDP-added water samples was evaluated.

The differences in response intensities between the four groups occur when the incubation temperature is near the minimum level studied and the sample volume is near the maximum level studied. According to the BBD-RSM model, the optimum conditions that resulted in the maximum discrimination between the four PDPs in water samples were as follows: 2.5 mL of sample, 5 min incubation time at 31 °C, with agitation at 750 rpm and a 0.91 mL injection volume.

The optimum incubation time and the incubation temperature were close to the lowest values studied. Shorter incubation times were not tested, as greater variations of the signal from the samples would be expected because of the headspace generation conditions. As regards the incubation temperature, no lower temperatures were allowed by the equipment used. On the other hand, the maximum discrimination was achieved at the highest levels within the range studied, which, on the other hand, were the maximum values allowed by the HS-GC-IMS system used.

### 3.2. Repeatability and Intermediate Precision of the Method

The repeatability and intermediate precision of the method were evaluated as explained below. The intermediate precision was determined as the proximity between the measured differences on different days and the repeatability was determined as the proximity between the differences measured in different experiments completed on the same day. A total of 12 experiments were conducted under the same optimum conditions on each one of the four sample types. Therefore, a total of 36 samples were analyzed as follows: 12 gasoline-added samples, 12 diesel-added samples, 12 lubricant-added samples, and 12 kerosene-added samples. All of them at 50 µL·L^−1^.

The coefficient of variation (CV) between the sum of intensity differences was calculated as a measurement of their similarities. The intermediate precision and repeatability of the method was calculated, and the results obtained were 2.35% and 3.09%, respectively. Both values were below the acceptable limit (5%). It was, therefore, considered that the repeatability and intermediate precision of the developed method was within acceptable limits.

### 3.3. Analysis of the PDPs in Water Samples

Once the method had been optimized, the applicability of this technique to discriminate between different PDPs in water samples had to be evaluated. Although 50 µL·L^−1^ concentration levels had to be used for optimization of the method, lower concentration levels should be used to validate the method and the highest PDP/water concentration level allowed by Spanish legislation, which is 8 µL·L^−1^, was included. In fact, not only the PDPs in the water samples had to be successfully discriminated, but the method should also prove its suitability to detect the presence of such PDPs in water at much lower concentrations. Two replicas of pure seawater samples were analyzed to obtain a homogeneous group.

The first samples to be analyzed were those with the maximum PDP content level allowed by Spanish law. A total of 18 samples (two replicas of pure seawater with the addition of any of the 16 PDPs included in Table 2 at 8 µL·L^−1^) were analyzed by HS-GC-IMS, according to the previously established optimum conditions. The IMSS of each one of the PDP-added samples was obtained and reduced to the drift range of interest (1.020 to 2.000 (RIP relative)). Then, the results were normalized to the maximum. Specific chemometric tools were applied to determine if the differences between IMSS responses could be used to successfully discriminate between the different PDPs added to the water samples.

**Figure 4:**
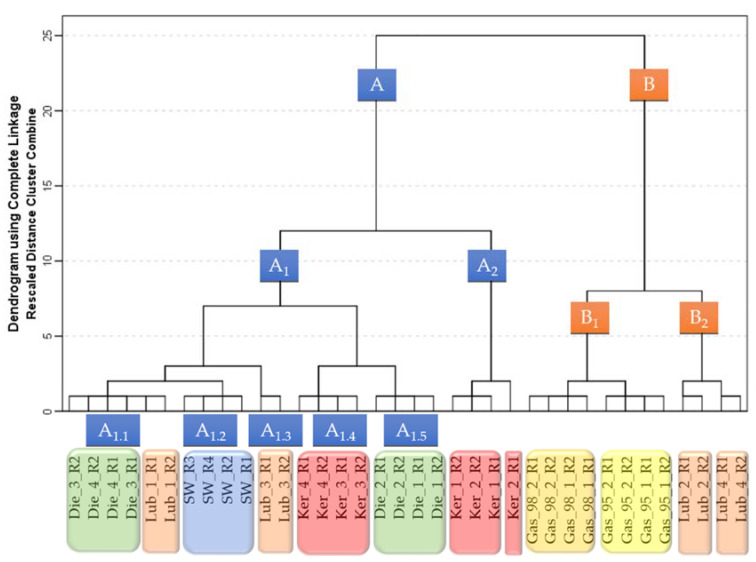
Dendrogram obtained by the hierarchical cluster analysis (HCA) of the pure water and the PDP-added samples (D_36X980_).

Some clear trends in the IMSS response differences could be visually observed. Thus, a non-supervised method was selected to evaluate such trends and to classify them according to the presence or absence of the PDP in the water and even according to the type of PDP. A HCA along with the furthest neighbor method and squared Euclidean distance was applied. The IMSS of the two replicas of each of the 18 samples were analyzed using this HCA method. The results were graphically represented in a dendrogram (Figure 4).

Two clear groups could be observed (A and B). Group B is divided into two clusters (B_1_ and B_2_). Cluster B_1_ comprises all the samples containing gasoline and a tendency to further classify them according to gasoline octane grade was observed. Cluster B_2_ includes four out of the eight lubricant-added samples.

Group A was also divided into two clusters (A_1_ and A_2_). Cluster A_1_ was formed by five subclusters. Subcluster A_1.1_ contains four out of the eight diesel-added samples, and two of the eight lubricant-added samples. Subcluster A_1.2_ was exclusively formed by all of the pure water samples. Subcluster A_1.3_ includes the remaining two lubricant-added samples. Subcluster A_1.4_ was solely formed by four of the eight kerosene-added samples, while subcluster A_1.5_ contains the remaining four diesel-added samples. Finally, Cluster A_2_ grouped together the four remaining kerosene-added samples.

The suitability of the method to detect PDP contamination was confirmed, since a clear tendency to classify the samples according to the presence or absence of PDPs could be observed. Furthermore, even though some misclassifications of PDPs with similar compositions could be observed, a tendency to group the samples according to the type of PDP was revealed, particularly in the case of gasoline.

In order to determine the drift time zones that were relevant for the discrimination of the samples, so that a total and complete discrimination between the samples could be achieved, a supervised technique, i.e., LDA was used. All of the 36 IMSSs were used for this purpose and 67% of them were selected to create the model, whereas the rest of the IMSSs were used to validate it. By means of the stepwise method, five groups were created as follows: pure water, gasoline-added, diesel-added, lubricant-added and kerosene-added samples.

**Figure 5:**
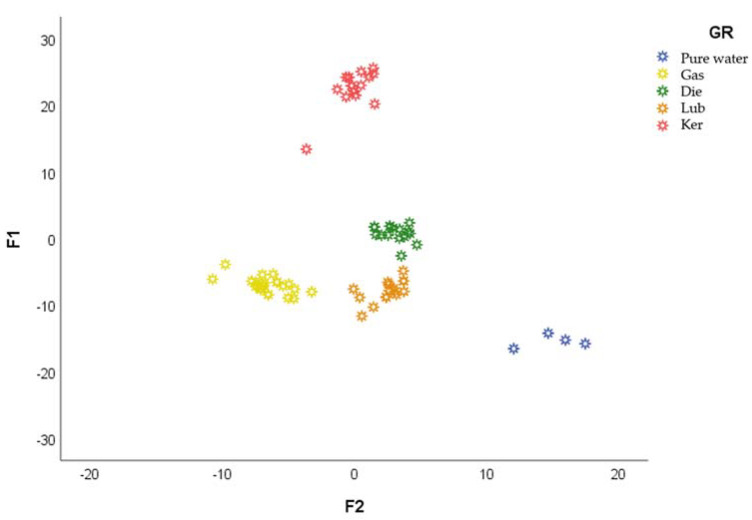
Graphic representation of the linear discriminant analysis (LDA) (D_36X980_) F_1_ and F_2_ values of all the samples.

All (100%) of the groups were successfully classified. The samples are represented in Figure 5, according to their first two discriminant functions (F1 and F2). It can be observed that F_1_, with positive scores, is the main responsible for the discrimination of the kerosene-added samples. Diesel-added samples scored around 0 for F_1_, while the rest of the samples presented negative scores. On the other hand, F_2_ allowed for the discrimination of the pure water samples, with positive scores, from the rest of the samples, with scores of almost zero or even negative scores in the case of gasoline-added samples. It can be seen that based on the IMS spectroscopic data, the lubricant-added samples and the diesel-added samples presented the closest values between them. Based on the LDA results, the PDPs could be detected and even identified according to their PDP type.

In order to test the suitability of the developed method, the PDP maximum content (8 µL·L^−1^) currently established by Spanish legislation as representing non-contaminated seawater was included. Therefore, the suitability of the developed method to detect and discriminate PDPs in water samples at a concentration of 8 µL·L^−1^ was tested and confirmed. Then, the method was tested for lower concentration levels. For that purpose, 64 PDP-altered samples were elaborated by adding each one of the 16 PDPs described in Table 2 at concentrations of 4 µL·L^−1^, 2 µL·L^−1^, 0.8 µL·L^−1^, and 0.4 µL·L^−1^. The samples were analyzed and each one of their IMSSs were reduced.

An LDA was applied to the samples with the same concentration level in order to verify if pure water and PDP-added samples could be successfully discriminated. Although all the samples containing PDPs at concentrations of 4 µL·L^−1^ and 2 µL·L^−1^ were fully and successfully discriminated, when their PDP concentration level went down to 0.8 µL·L^−1^, some of the lubricant or diesel-added samples were misclassified (Table 5). It was then observed that even at concentrations as low as 0.4 µL·L^−1^, 100% of the PDPs were successfully detected, with 100% discrimination between the pure water group and the rest of the groups. However, the misclassification between the different PDPs increased. Therefore, 2 µL·L^−1^—which represents 25% of the lowest legal limit in Spain—was established as the lowest concentration limit that this method can handle efficiently.

Considering such minimum concentration level requirements, only the samples containing any of the PDPs of interest at concentrations of 8 µL·L^−1^, 4 µL·L^−1^, and 2 µL·L^−1^ were used to elaborate the discrimination functions that would allow for the detection and identification of each PDP added to the water. Therefore, an LDA of pure water and PDP-added samples at concentrations of 8 µL·L^−1^, 4 µL·L^−1^, and 2 µL·L^−1^ was conducted. A total of 100 samples were analyzed using the stepwise cross-validation method. Successful classification of 96% of the samples was achieved. Only three diesel-added samples at 2 µL·L^−1^ were misclassified as lubricant-added samples, while one lubricant-added sample at 2 µL·L^−1^ was misclassified as a diesel-added sample. It could be observed that these errors took place at low concentration levels and between groups with similar PDP compositions. The resulting model, i.e., five discrimination functions for water, Gas, Die, Lub and Ker, will allow for the detection of PDP in water samples at concentration levels between 2 µL·L^−1^ and 8 µL·L^−1^.

### 3.4. Application to Natural Samples

Once the suitability of the method for the detection and discrimination of PDP-added samples at concentrations as low as 2 µL·L^−1^ was tested and confirmed, the method was tested against unaltered seawater samples (Table 2). The five discriminant functions previously obtained were applied to the unaltered seawater sample IMSS response intensities at the drift times that had been selected for the pure samples. Seven of the unaltered seawater samples were collected and analyzed in duplicate and most of the samples were classified in the pure water group. Nevertheless, the samples from Algeciras and Cadiz ports showed IMSS response intensities that agreed with those previously obtained for diesel and lubricant-added water samples. This suggests that diesel and lubricant were present in the samples at concentration levels higher than 2 µL·L^−1^.

## 4. Conclusions

In the present study, a method based on HS-IMS—in which IMS was used as a multiple sensor device to discriminate PDPs in water samples—was optimized. According to our results, the optimum conditions were 2.5 mL incubated for 5 min at 31 °C and agitated at 750 rpm. The optimized method exhibited good repeatability and intermediate precision with RSDs lower than 5% for both factors.

The developed method was confirmed as being suitable for the discrimination of any of the 16 PDPs included in this study and added to seawater samples at concentrations as low as 2 µL·L^−1^. Naturally, full discrimination between pure seawater and PDP-added samples was also achieved at concentrations as low as 0.4 µL·L^−1^, which represents 5% of the maximum concentration allowed by Spanish legislation.

In summary, the developed method based on HS-GC-IMS, in which IMSS is used in combination with a number of chemometric tools, has proven to be a practical approach that can be employed in environmental forensic investigations for the detection and discrimination of PDPs in seawater.

## Figures and Tables

**Table 1:** Natural seawater samples collected to optimize the analytical method: location and identification acronym included.

Acronym	Location
RS_1	Seawater sample from El Rinconcillo Beach. Algeciras. Spain (36°09’48.7” N 5°26’22.8” W). Collected on: 28/02/2018.
RS_2	Seawater sample from the port near El Rinconcillo Beach. Algeciras. Spain (36°09’45.1” N 5°26’06.9” W). Collected on: 13/01/2018.
RS_3	Seawater sample taken between grounded boats. La Caleta Beach. Cadiz. Spain (36°31’54.7” N 6°18’21.9” W). Collected on: 14/03/2018.
RS_4	Seawater sample from Punta Candor Beach. Cadiz. Spain (36°38’32.1” N 6°23’34.5” W). Collected on: 29/01/2018.
RS_5	Seawater sample from La Calita Beach in Puerto Sherry. El Puerto de Santa Maria. Spain (36°34’60.0” N 6°16’05.1” W). Collected on: 15/04/2018.
RS_6	Seawater sample from Puerto Sherry. El Puerto de Santa María. Spain (36°34’50.0” N 6°15’03.4” W). Collected on: 08/02/2018.
RS_7	Seawater sample from Cadiz harbor. Cadiz. Spain (36°32’01.6” N 6°17’31.5” W). Collected on: 23/03/2018.

**Table 2:** Petroleum-derived products used for this research.

Group	Acronym	Description
Gasoline (Gas)	Gas_95_1	Gasoline 95 octane. Collected on: 28/06/2018. REPSOL Gas Station El Puerto de Santa Maria. Spain.
	Gas_95_2	Gasoline 95 octane. Collected on: 29/06/2018. Carrefour Gas Station Jerez de la Frontera. Spain.
	Gas_98_1	Gasoline 98 octane. Collected on: 19/09/2018. REPSOL Gas Station Cordoba. Spain.
	Gas_98_2	Gasoline 98 octane. Collected on: 20/07/2018. Carrefour Gas Station Jerez de la Frontera. Spain.
Diesel (Dies)	Dies_1	Automotive diesel fuel (A)-diesel e+ neotech. Collected on: 27/05/2018. REPSOL Gas Station Torre del Mar. Spain.
	Dies_2	Automotive diesel fuel (A)-diesel e+ neotech. Collected on: 20/06/2018. REPSOL Gas Station Torre del Mar. Spain.
	Dies_3	Industrial diesel fuel (B)-diesel e+. Collected on: 05/05/2018. REPSOL Gas Station Port Caleta de Velez. Spain.
	Dies_4	Industrial diesel fuel (B)-diesel e+. Collected on: 10/06/2018. CEPSA Gas Station Port Caleta de Velez. Spain.
Lubricant (LUB)	LUB_1	Engine lubricant 2T. Cepsa Store. Spain. Collected on: 17/05/2018.
	LUB_2	Engine lubricant 2T. Racing Store. Spain. Collected on: 10/08/2018.
	LUB_3	Boat lubricant. Cadiz Port. Spain. Collected on: 14/07/2018.
	LUB_4	Boat lubricant. Cadiz Port. Spain. Origin unknown. Collected on: 24/05/2018.
Kerosene (Ker)	Ker_1	Aviation kerosene. Collected on: 17/06/2018. Malaga airport. Spain.
	Ker_2	Aviation kerosene. Collected on: 25/07/2018. Malaga airport. Spain.
	Ker_3	Aviation kerosene. Collected on: 02/06/2018. Airfield La Axarquia-Leoni Benabu. Malaga. Spain.
	Ker_4	Aviation kerosene. Collected on: 22/07/2018. Airfield La Axarquia-Leoni Benabu. Malaga. Spain.

**Table 3:** Selected variable values and their coded and non-coded levels for the Box–Behnken design–response surface methodology (BBD-RSM).

Variable	−1	0	1
Incubation time (min)	5	15	25
Incubation temperature (°C)	30	50	70
Agitation (rpm)	250	500	750
Injection volume (mL)	0.5	0.75	1
Sample volume (mL)	0.5	1.5	2.5

**Table 4:** ANOVA of the quadratic model for the discrimination of the different petroleum-derived products (PDPs) in the water samples.

Variable	Factor	Coefficient	*F*-Value	*p*-Value
Incubation temperature	X_1_	−1294.850	107.740	0.000
Incubation time	X_2_	−85.321	0.470	0.500
Agitation	X_3_	65.092	0.270	0.606
Injection volume	X_4_	254.135	4.150	0.052
Sample volume	X_5_	1212.570	94.490	0.000
Incubation temperature: Incubation temperature	X_1_^2^	−284.903	2.850	0.104
Incubation temperature: Incubation time	X_1_X_2_	−47.107	0.040	0.852
Incubation temperature: Agitation	X_1_X_3_	−192.386	0.590	0.448
Incubation temperature: Injection volume	X_1_X_4_	−59.996	0.060	0.812
Incubation temperature: Sample volume	X_1_X_5_	−326.713	1.710	0.202
Incubation time: Incubation time	X_2_^2^	−102.797	0.370	0.548
Incubation time: Agitation	X_2_X_3_	−58.636	0.060	0.816
Incubation time: Injection volume	X_2_X_4_	−81.193	0.110	0.748
Incubation time: Sample volume	X_2_X_5_	−304.884	1.490	0.233
Agitation: Agitation	X_3_^2^	217.615	1.660	0.209
Agitation: Injection volume	X_3_X_4_	7.701	0.000	0.976
Agitation: Sample volume	X_3_X_5_	60.019	0.060	0.812
Injection volume: Injection volume	X_4_^2^	123.217	0.530	0.473
Injection volume: Sample volume	X_4_X_5_	−123.500	0.250	0.625
Sample volume: Sample volume	X_5_^2^	−554.015	10.760	0.003

**Table 5:** LDA data corresponding to the total and percentage of successful pure water and PDP-added sample classifications.

Concentration (µL·L^−1^)	Pure Water	Gas	Die	Lub	Ker
8	4 (100%)	8 (100%)	8 (100%)	8 (100%)	8 (100%)
4	4 (100%)	8 (100%)	8 (100%)	8 (100%)	8 (100%)
2	4 (100%)	8 (100%)	8 (100%)	8 (100%)	8 (100%)
0.8	4 (100%)	8 (100%)	7 (87.5%)	100 (100%)	8 (100%)
0.4	4 (100%)	8 (100%)	7 (87.5%)	7 (87.5%)	8 (100%)

## Data Availability

Data sharing not applicable.

## Appendix A

**Table A1:** Box-Behnken design matrix with the values of the six variables for each experiment and measured and predicted responses.

Experiment	Incubation Time (min)	Incubation Temperature (°C)	Agitation (rpm)	Injection Volume (mL)	Sample Volume (mL)	Measured Response	Predicted Response	Relative Error (%)
1	15	50	750	0.75	0.50	1995.15	1927.07	3.47
2	15	50	250	0.50	1.50	3136.79	2913.67	7.38
3	15	50	500	0.75	1.50	3006.22	2999.02	0.24
4	15	50	500	1.00	0.50	2072.53	2066.15	0.31
5	15	50	250	0.75	0.50	1838.73	1922.00	4.43
6	15	50	750	0.50	1.50	3059.73	2771.06	9.90
7	15	30	500	1.00	1.50	3432.70	3422.67	0.29
8	15	50	750	1.00	1.50	3022.98	3032.90	0.33
9	5	50	500	1.00	1.50	2975.55	2919.55	1.90
10	15	50	500	0.50	0.50	1648.85	1688.52	2.38
11	15	50	250	1.00	1.50	3084.64	2960.11	4.12
12	15	30	250	0.75	1.50	3287.25	3184.06	3.19
13	25	50	750	0.75	1.50	2736.63	2717.00	0.72
14	5	50	500	0.75	0.50	1588.39	1654.55	4.08
15	25	50	250	0.75	1.50	2640.63	2710.54	2.61
16	5	50	500	0.50	1.50	2637.91	2584.23	2.06
17	15	70	750	0.75	1.50	1778.89	1954.30	9.40
18	15	50	500	0.75	1.50	2874.66	2699.02	6.30
19	15	50	500	0.75	1.50	2874.66	2699.02	6.30
20	25	70	500	0.75	1.50	1786.34	1791.53	0.29
21	5	50	750	0.75	1.50	2759.58	2860.95	3.61
22	25	50	500	0.50	1.50	2366.48	2580.10	8.64
23	15	70	500	1.00	1.50	2189.53	2067.82	5.72
24	5	70	500	0.75	1.50	1715.84	1723.96	0.47
25	15	30	500	0.75	2.50	3472.08	3696.63	6.26
26	5	50	500	0.75	2.50	3517.25	3472.00	1.29
27	15	70	250	0.75	1.50	2012.62	2081.60	3.37
28	5	30	500	0.75	1.50	3283.33	3171.70	3.46
29	15	70	500	0.75	2.50	2230.74	2075.06	7.23
30	15	50	500	0.75	1.50	2454.59	2699.02	9.49
31	15	50	500	1.00	2.50	3057.82	3155.22	3.14
32	25	50	500	0.75	0.50	1751.32	1874.11	6.77
33	25	30	500	0.75	1.50	3448.04	3133.49	9.56
34	25	50	500	1.00	1.50	2541.73	2753.04	7.98
35	15	50	250	0.75	2.50	2836.79	3074.55	8.04
36	15	30	500	0.75	0.50	2085.95	2157.35	3.37
37	25	50	500	0.75	2.50	3070.42	2781.79	9.86
38	15	50	750	0.75	2.50	3113.24	3199.66	2.74
39	15	70	500	0.50	1.50	1745.15	1873.68	7.10
40	15	50	500	0.75	1.50	2361.75	2499.02	5.65
41	15	30	500	0.50	1.50	2868.33	3108.54	8.04
42	15	30	750	0.75	1.50	3438.30	3441.54	0.09
43	15	50	500	0.50	2.50	2881.15	3024.59	4.86
44	15	70	500	0.75	0.50	1498.05	1489.21	0.59
45	15	50	500	0.75	1.50	2622.25	2699.02	2.89
46	5	50	250	0.75	1.50	2546.31	2737.23	7.23
